# Carbon–TiO_2_ Hybrid Quantum Dots for Photocatalytic Inactivation of Gram-Positive and Gram-Negative Bacteria

**DOI:** 10.3390/ijms25042196

**Published:** 2024-02-12

**Authors:** Xiuli Dong, Yamin Liu, Audrey F. Adcock, Kirkland Sheriff, Weixiong Liang, Liju Yang, Ya-Ping Sun

**Affiliations:** 1 Department of Pharmaceutical Sciences, Biomanufacturing Research Institute and Technology Enterprise, North Carolina Central University, Durham, NC 27707, USA; xdong@campbell.edu (X.D.); lyang@nccu.edu (L.Y.); 2 Department of Microbiology and Immunology, School of Osteopathic Medicine, Campbell University, Buies Creek, NC 27506, USA; 3 Department of Chemistry, Clemson University, Clemson, SC 29634, USA

**Keywords:** carbon dots, carbon–TiO_2_ hybrid dots, antibacterial function, Gram-negative bacteria, fluorescence performance, photosensitization

## Abstract

Carbon–semiconductor hybrid quantum dots are classical carbon dots with core carbon nanoparticles doped with a selected nanoscale semiconductor. Specifically, on those with the nanoscale TiO_2_ doping, denoted as C_TiO2_-Dots, their synthesis and thorough characterization were reported previously. In this work, the C_TiO2_-Dots were evaluated for their visible light-activated antibacterial function, with the results showing the effective killing of not only Gram-positive but also the generally more resistant Gram-negative bacteria. The hybrid dots are clearly more potent antibacterial agents than their neat carbon dot counterparts. Mechanistically, the higher antibacterial performance of the C_TiO2_-Dots is attributed to their superior photoexcited state properties, which are reflected by the observed much brighter fluorescence emissions. Also considered and discussed is the possibility of additional contributions to the antibacterial activities due to the photosensitization of the nanoscale TiO_2_ by its doped core carbon nanoparticles.

## 1. Introduction

**Figure 1:**
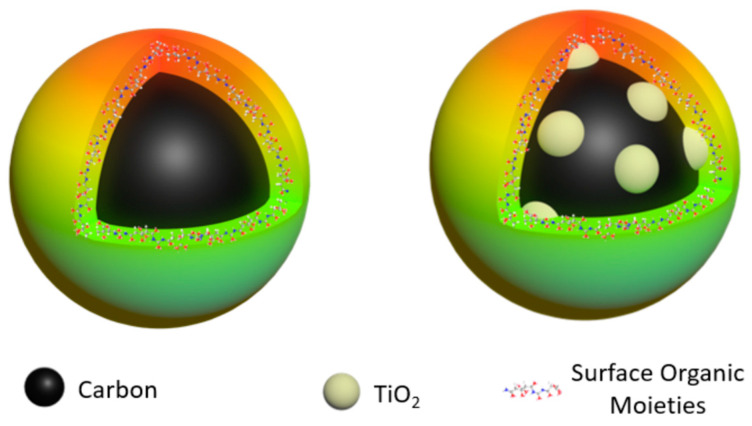
Cartoon illustration of (**left**) classical CDots in the structure of a small carbon nanoparticle core with surface organic functionalization and (**right**) the carbon–TiO_2_ hybrid dots, C_TiO2_-Dots, in which the small carbon nanoparticle core is doped with nanoscale TiO_2_ and also with the same organic functionalization.

Small carbon nanoparticles (CNPs) as nanoscale carbon allotropes at zero-dimension, joining the one-dimensional carbon nanotubes and two-dimensional graphenes, have attracted rapidly increasing attention in the recent literature [1,2,3]. The properties of CNPs including, especially, their optical and photoexcited state properties could be dramatically enhanced when the CNP surface is passivated effectively via the deliberate chemical functionalization of organic species, with the resulting surface-functionalized CNPs defined as carbon “quantum” dots or carbon dots (CDots, Figure 1) [1,4,5]. CDots are essentially special core–shell nanostructures each with a CNP core and a corona-like soft shell of organic species dominated by those chemically bonded to the CNP core (Figure 1). The most visible property enhancement from CNPs to CDots is that the latter exhibits bright and colorful fluorescence emissions, while “naked” CNPs in solvent dispersions are in general only weakly emissive [6]. The brightly fluorescent CDots are also known for their potent antimicrobial activities with visible light exposure [7,8,9,10,11,12,13,14]. The photoinduced antimicrobial properties of CDots may also share some mechanistic features with those of classical semiconductor quantum dots (QDs) [15,16,17,18].

It has also been demonstrated that CNPs are compatible with nanoscale semiconductors for carbon-based/derived hybrid “quantum” dots. Among the hybrid dot configurations more relevant to the work reported here are those from the doping or coating of CNPs with nanoscale semiconductors such as ZnS, ZnO, or TiO_2_ and then the same organic functionalization as that in the preparation of neat CDots, and the resulting carbon–semiconductor hybrid “quantum” dots are denoted as C_ZnS_-Dots, C_ZnO_-Dots, or C_TiO2_-Dots, respectively [19,20]. These hybrid dots have been found to exhibit greatly enhanced photoexcited state properties compared to those of their neat CDots counterparts, with most having visibly much brighter fluorescence emissions and correspondingly higher observed fluorescence quantum yields. 

The photoexcited state properties of CDots are known to dictate their light-activated antimicrobial function. Thus, it should be expected that the enhancement of such properties in the carbon-based/derived hybrid dots would result in their improved antimicrobial performance. Indeed, it was found in the work reported here that the C_TiO2_-Dots with exposure to visible light are very effective in killing not only Gram-positive bacteria but also the generally more resistant Gram-negative bacteria. The C_TiO2_-Dots represent an interesting platform of carbon-based/derived hybrid dots, with superior optical spectroscopy properties. The findings on their similarly superior photoinduced antimicrobial function in the study reported here demonstrate the excellent potential of the carbon–semiconductor hybrid dots, opening up a new frontier in the development of high-performance antimicrobial nanomaterials.

## 2. Results 

**Figure 2:**
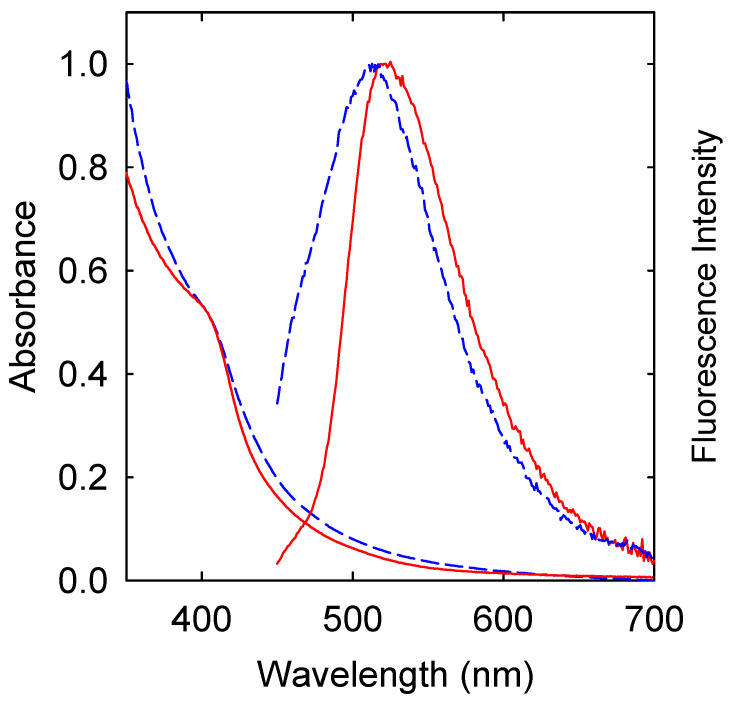
Absorption and fluorescence (440 nm excitation) spectra of C_TiO2_-Dots (solid line) and PEG_1500N_-CDots (dashed line) in aqueous solutions.

The preparation and characterization of C_TiO2_-Dots have been established in previously reported studies [20]. The CNPs were harvested from the oxidative acid-treated carbon nanopowder sample, and the known carboxylic acid moieties on the surfaces of CNPs benefited their more homogenous dispersion in the ethanol–water–nitric acid mixture. In the same solvent mixture, the organo-titanium compound Ti(OC_2_H_5_)_4_ was hydrolyzed to form Ti(OH)_4_, with a preference on the surfaces of the dispersed CNPs due to nucleation effects, followed by their dehydration to become TiO_2_ in the subsequent thermal annealing process. The resulting TiO_2_-doped CNPs were treated with *O*,*O*’-bis(3-aminopropyl) polyethylene glycol of average molecular weight ~1500 (PEG_1500N_), which was designed to target those doped CNPs still of some surface-bound carboxylic acid moieties to form zwitterionic bonds. The PEG_1500N_ functionalization of the TiO_2_-doped CNPs yielded C_TiO2_-Dots. These carbon–TiO_2_ hybrid dots are CNPs each with the surface effectively passivated by the combination of TiO_2_ doping and PEG_1500N_ functionalization. Such a structural configuration is consistent with the available results from the transmission electron microscopy imaging at high resolution [19,20]. Some microscopy characterization results are provided in the Supplementary Materials. However, the XRD probing of the TiO_2_ domains in the hybrid dot sample did not yield useful information due to the low TiO_2_ content and probably more so to the severe signal broadening effects associated with the ultra-nanoscopic TiO_2_ domain sizes. The effectiveness of the combined surface passivation effects was reflected by the observed very bright fluorescence emissions with correspondingly high fluorescence quantum yields (Φ_F_). The Φ_F_ value of the C_TiO2_-Dots sample used in the antibacterial experiments was 41%, versus the Φ_F_ value of 10–12% for the similarly synthesized PEG_1500N_-CDots sample without any TiO_2_ doping. However, the optical absorption and fluorescence emission spectral features of C_TiO2_-Dots and PEG_1500N_-CDots in solutions were not different in any dramatic fashion (Figure 2) [21].

**Figure 3:**
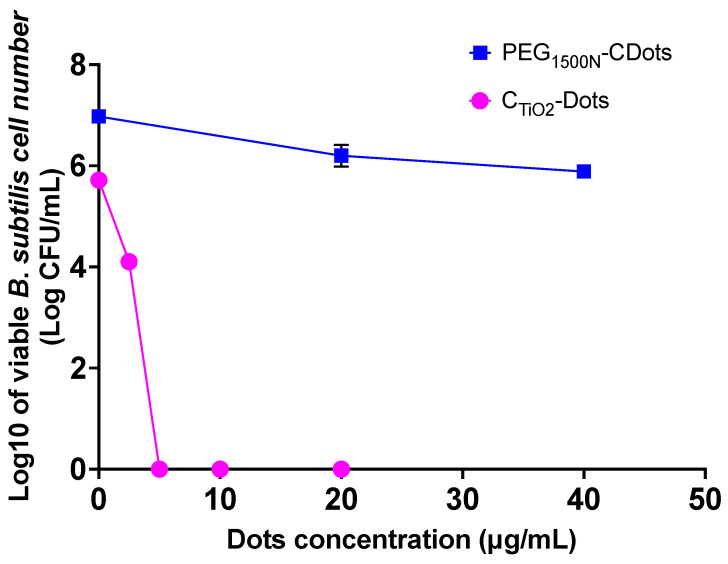
The dose–response curves of C_TiO2_-Dots and PEG_1500N_-CDots with 2 h visible light exposure for the inactivation of *B. subtilis* cells.

CDots of different surface organic functionalizations with visible light exposure have exhibited potent antibacterial activities against Gram-positive bacteria [8]. In the evaluation experiments using the Gram-positive *B. subtilis* as the target, the bacterial cells in PBS suspensions (~10^6^–10^7^ CFU/mL) were treated with C_TiO2_-Dots at different concentrations under visible light exposure for 2 h. After the treatments, the viable cell numbers in the treated samples and the control samples were determined. As shown in Figure 3, in the dose–response curves of *B. subtilis*, the C_TiO2_-Dots sample with visible light exposure is clearly highly efficient in inactivating *B. subtilis*, such that the treatment with 5 μg/mL C_TiO2_-Dots can completely inactivate all cells in the tested bacterial sample corresponding to ~6 log viable cell reduction in *B. subtilis* cells.

For comparison, the antibacterial activity against *B. subtilis* by the similarly structured PEG_1500N_-CDots but without TiO_2_ doping was evaluated under the same light exposure and test conditions. According to the dose–response curve also shown in Figure 3, the visible light-activated antibacterial function of the PEG_1500N_-CDots was much weaker, such that the treatment with 20 μg/mL of the dot sample resulted in only ~0.8 log viable cell reduction in *B. subtilis*. The effect of increasing the concentration of the CDots was not so significant either, with the doubling of the PEG_1500N_-CDots concentration to 40 μg/mL in the treatment resulting in only slightly increased viable cell reduction in *B. subtilis* to ~1.1 log (Figure 3).

**Figure 4:**
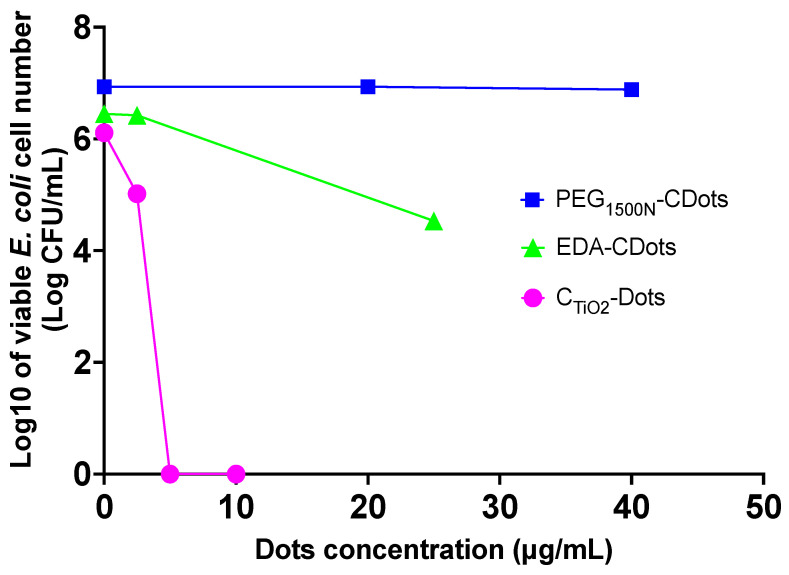
The dose–response curves of C_TiO2_-Dots, PEG_1500N_-CDots, and EDA-CDots with 2 h visible light exposure for the inactivation of *E. coli* cells.

Gram-negative bacteria are generally more resistant to many antibiotics and antimicrobial agents due to the permeability barrier properties of their outer membrane [22,23,24,25,26,27]. Such a membrane containing lipopolysaccharide represents a unique structural feature of Gram-negative bacteria, and makes it more difficult for antibiotics and antibacterial agents to penetrate and reach the cytoplasm for action [28,29,30,31,32,33,34,35]. In fact, combating Gram-negative bacteria has been a historic challenge in the antimicrobial research field [36,37,38,39,40,41,42]; so, any success in developing antimicrobial agents capable of effectively inactivating Gram-negative bacteria is highly valuable. In this study, *E. coli* was selected as a representative of Gram-negative bacteria for the evaluation of the antibacterial activities of C_TiO2_-Dots and PEG_1500N_-CDots with visible light exposure. The experimental conditions for the evaluations were similar to those for *B. subtilis* discussed above. The *E. coli* cells in PBS suspensions (~10^6^–10^7^ CFU/mL) were treated with C_TiO2_-Dots at different concentrations with visible light exposure for 2 h, followed by the determination of the viable cell numbers in the treated samples and the controls. As shown in Figure 4, the C_TiO2_-Dots sample with visible light exposure is highly efficient in inactivating Gram-negative *E. coli* cells, with the effectiveness comparable to that against *B. subtilis*. More quantitatively, the treatment with 2.5 μg/mL C_TiO2_-Dots resulted in ~1.1 log viable cell reduction, and a higher C_TiO2_-Dots concentration of 5 μg/mL could completely inactivate all *E. coli* cells in the tested bacterial samples, corresponding to ~6 log viable cell reduction. 

For comparison, the PEG_1500N_-CDots sample was used to treat *E. coli* cells with the same visible light exposure, and the results are shown in Figure 4. Obviously, the PEG_1500N_-CDots sample with visible light was incapable of inactivating *E. coli* cells, even with the much higher dot concentrations of 20–40 μg/mL (Figure 4).

Among the classically defined and synthesized CDots, which are simply small carbon nanoparticles (CNPs) with deliberate chemical functionalization of the nanoparticle surface by organic species [1], EDA-CDots [EDA = 2,2′-(ethylenedioxy)bis(ethylamine)] [43] have been considered a benchmark dot sample for their generally potent visible light-activated antimicrobial function, including their ability to inactivate Gram-negative bacteria like *E. coli* [7,44]. However, as also shown by the comparison in Figure 4, the C_TiO2_-Dots are still significantly more effective. A clear mechanistic understanding on the effectiveness of C_TiO2_-Dots is beyond the scope of this work, but some considerations that might be relevant to the mechanistic origins of the observed different antibacterial behaviors are as follows.

## 3. Discussion

In general, the photoexcited state properties of CDots, which ultimately drive their antimicrobial activities, are correlated with their observed fluorescence parameters, more specifically, fluorescence quantum yields (Φ_F_). There is experimental evidence for the positive correlation between the antimicrobial effectiveness of the CDots and their observed fluorescence quantum yields [45], which are further correlated with the effectiveness of the surface functionalization of the core CNPs in the CDots [1]. Therefore, it may be argued that the poor performance of the PEG_1500N_-CDots against *E. coli* might be attributed to their observed relatively low fluorescence quantum yields due to the less effective functionalization of the core CNPs by PEG_1500N_ via the zwitterionic bonding. Consistent with such an argument is the generally more effective functionalization of the core CNPs in the EDA-CDots, with higher observed fluorescence quantum yields [43]. For the C_TiO2_-Dots, the high fluorescence quantum yields due to the combined surface passivation by the TiO_2_ doping and PEG_1500N_ attachment may be used to account for the high performance in the visible light-driven inactivation of Gram-negative *E. coli*, though there could also be other contributing factors. In the C_TiO2_-Dots, while the nanoscale TiO_2_ moieties are not absorptive in the visible spectral region, such moieties are known to have the capability for photosensitization due to photoexcited molecular dyes with absorptions in the visible light spectrum [46,47,48,49,50]. It is possible that there is photosensitization of the nanoscale TiO_2_ moieties by the nanocarbon part of C_TiO2_-Dots upon visible light excitation, which could contribute to the overall antibacterial activities. This is obviously a complicated structure–property relationship issue that deserves further dedicated investigations.

## 4. Material and Methods

### 4.1. Materials

The carbon nanopowder sample was acquired from US Research Nanomaterials, Inc. (Houston, TX, USA). *O*,*O*’-Bis(3-aminopropyl) polyethylene glycol (PEG_1500N_, average molecular weight ~1500) was purchased from Aldrich, Ti(OC_2_H_5_)_4_ (>97%) from Alfa Aesar, and sodium dodecyl sulfate (SDS, 99%), nitric acid (60–70%), and ethanol (>99%) from VWR. Dialysis membrane tubing was obtained from Spectrum Laboratories. Water was deionized and purified by using a Labconco WaterPros water purification system (Labconco, Kansas City, MO, USA).

### 4.2. Measurement

UV/vis absorption spectra were recorded on a Shimadzu UV2501-PC spectrophotometer. Fluorescence spectra were measured on a Jobin-Yvon emission spectrometer equipped with a 450 W xenon source, Gemini-180 excitation, Triax-550 emission monochromators, and a photon-counting detector (Hamamatsu R928P PMT at 950 V). 9,10-Bis(phenylethynyl)-anthracene in cyclohexane was used as a standard in the determination of fluorescence quantum yields through the relative method (matching the absorbance at the excitation wavelength between the sample and standard solutions and comparing their corresponding integrated total fluorescence intensities). X-ray diffraction measurements were performed on a Rigaku Ultima IV X-ray diffractometer with Cu Kα radiation (λ = 1.5418 Å).

### 4.3. C_TiO2_-Dots and PEG_1500N_-CDots

The carbon nanopowder sample (1 g) was refluxed in an aqueous nitric acid solution (5 M, 100 mL) for 24 h. The acidic suspension from the processing was cooled to room temperature and centrifuged at 1000× *g* to retain the supernatant, which was then dialyzed (molecular weight cut-off ~500) against fresh water. The resulting aqueous suspension was evaporated to remove water to obtain a sample of small carbon nanoparticles (CNPs).

The preparation of C_TiO2_-Dots followed the procedure and conditions reported previously [20]. Briefly, a clear solution of Ti(OC_2_H_5_)_4_ (2.9 g) in a mixture of ethanol (51 mL), water (0.43 mL), and nitric acid (0.16 mL) was prepared. To an aliquot (50 mL) of the solution was added the CNPs (200 mg), and the mixture was sonicated for 1 h, stirred for 12 h, and then filtrated. The filter cake was grounded and annealed at 250 °C for 1 h to obtain a solid sample. A portion (50 mg) of the sample was dispersed in an aqueous sodium dodecyl sulfate (SDS) solution (1 wt%, 120 mL) with sonication for 30 min, followed by filtration. The filter cake was washed with water repeatedly, and then dried. The solid sample thus obtained was mixed well with PEG_1500N_ (1 g), and the mixture was heated to 110 °C and stirred for 72 h under nitrogen protection. The reaction mixture was cooled back to ambient temperature and dispersed in water (15 mL). The dispersion was centrifuged at 20,000× *g* to retain the supernatant as an aqueous solution of the PEG_1500N_-C_TiO2_-Dots (denoted simply as C_TiO2_-Dots throughout this report).

For PEG_1500N_-CDots, a sample of the CNPs without the treatment for TiO_2_ doping was mixed well with PEG_1500N_, and the mixture was heated to 110 °C and stirred for 72 h under nitrogen protection. The reaction mixture was allowed to cool back to ambient temperature and then dispersed in water, followed by centrifugation at 20,000× *g* to retain the supernatant as an aqueous solution of the dot sample.

The details of the synthesis and characterization of EDA-CDots, which are small carbon nanoparticles with 2,2′-(ethylenedioxy)bis(ethylamine) (EDA) for surface functionalization, have been reported previously [43].

### 4.4. Bacterial Strains and Cultures

*B. subtilis* and *E. coli* K12 cultures were grown in 10 mL nutrient broth (Becton, Dickinson and Company, Sparks, MD, USA) by inoculating the broth with a single colony of a plated culture on a Luria–Bertani (LB) agar (Fisher Scientific, Fair Lawn, NJ, USA) plate, and incubated overnight at 37 °C. The freshly grown *B. subtilis* and *E. coli* cells were washed twice with phosphate-buffered saline (PBS, 1X, pH 7.4) (Fisher Scientific, Pittsburgh, PA, USA) and then resuspended in PBS for experimental uses.

### 4.5. Treatment of Bacterial Cells

The treatment of bacterial cells (*B. subtilis* or *E. coli*) with C_TiO2_-Dots, PEG_1500N_-CDots, or EDA-CDots was performed in 96-well plates. Aliquots of 150 µL of bacteria cell suspension and 50 µL of the selected dot sample at desired concentrations were placed into each well. The final bacterial cell concentration in each well was about ~10^6^–10^7^ CFU/mL, and the final concentration of the tested dots varied from 2 to 200 μg/mL. All samples were triplicated. The plate was placed on an orbital shaker (BT Lab Systems, St. Louis, MO, USA), with shaking at 300 rpm, and exposed to visible light from a commercially acquired household 60 W-equivalent daylight LED bulb (CREE, omnidirectional 815 lumens) placed at ~10 cm above the surface of the plate for 2 h. 

### 4.6. Assessment of Antibacterial Activity

After the treatments of the dot samples with visible light exposure, the viable cell numbers in the treated samples and the control samples were determined by the traditional surface plating method. Briefly, the bacterial samples were serially diluted (1:10) with PBS. Aliquots of 100 μL appropriate dilutions were surface-plated on LB agar plates. After incubation at 37 °C for 24 h, the colonies on the plates were counted, and the viable cell numbers were calculated in terms of the colony-forming units per milliliter (CFU/mL) for all of the treated samples and the controls. The logarithmic values of the viable cell numbers in the samples were plotted against the dot concentrations used in the treatments to generate dose-dependent curves for the different dot samples. The reduction in the logarithmic value of viable cell number in the treated samples in comparison to the controls (without any dots) was used to evaluate the antibacterial activities of the C_TiO2_-Dots, PEG_1500N_-CDots, and EDA-CDots. Under the defined concentration/conditions, the greater the viable cell reduction, the more potent the antibacterial activity of the dot sample.

## 5. Conclusions

In summary, C_TiO2_-Dots may be considered as hybrid CDots in which the more effective surface passivation of the core CNPs is achieved by a combination of the core nanoparticle surface doping with nanoscale TiO_2_ and organic (PEG_1500N_) functionalization, resulting in much brighter fluorescence emissions and higher quantum yields than those of the corresponding PEG_1500N_-CDots without the TiO_2_ doping or even the benchmark dot sample EDA-CDots. Equally superior are the photoinduced antibacterial activities of C_TiO2_-Dots over those of the neat CDots, with the particularly noticeable performance in the inactivation of the more resistant Gram-negative bacteria. Thus, the carbon-based/derived hybrid dots, with the C_TiO2_-Dots representing an interesting and effective platform, offer excellent opportunities for the development of visible light-driven antimicrobial agents capable of inactivating Gram-negative bacteria and other more resistant pathogens. Mechanistically, the photoexcited state properties of CDots are responsible for their antimicrobial functions. With the known positive correlation between fluorescence quantum yields and antimicrobial performances of CDots, one may credit the observed more effective antibacterial action of C_TiO2_-Dots to the high fluorescence quantum yields of the hybrid CDots. Nevertheless, it remains an interesting and fundamentally important question as to whether there is photosensitization of the nanoscale TiO_2_ doped on the surface of core CNPs in the hybrid CDots and the associated additional contributions to the observed high antibacterial performance.

## Data Availability

All data is contained within the article or Supplementary Material.

## Supplementary Materials

The following supporting information can be downloaded at: https://www.mdpi.com/article/10.3390/ijms25042196/s1.
