# Supplementary material for: Carbon–TiO2 Hybrid Quantum Dots for Photocatalytic Inactivation of Gram-Positive and Gram-Negative Bacteria
Source: Int J Mol Sci. 2024 Feb 12;25(4):2196. doi: 10.3390/ijms25042196 (PMC10889188; doi:10.3390/ijms25042196)
Supplement: Supplementary file 1 [file ijms-25-02196-s001.zip › ijms-2812168-supplementary.pdf]

Carbon-TiO_2 Hybrid Quantum Dots for Photocatalytic Inactivation of Gram-Positive and Gram-Negative Bacteria

Xiuli Dong [1,2], Yamin Liu [3], Audrey F. Adcock [1], Kirkland Sheriff [3], Weixiong Liang [3], Liju Yang [1] and Ya-Ping Sun [3,*]

[1]*Department of Pharmaceutical Sciences, Biomanufacturing Research Institute and Technology Enterprise, North Carolina Central University, Durham, NC 27707, USA*

[2]*Department of Microbiology and Immunology, School of Osteopathic Medicine, Campbell University, Buies Creek, NC 27506, USA*

[3]*Department of Chemistry, Clemson University, Clemson, SC 29634, USA*

[*] Correspondence: syaping@clemson.edu.

Supplementary Materials

Transmission electron microscopy (TEM) images shown in Figure SM1 were obtained on Hitachi 9500 high-resolution TEM system. In the preparation of specimen for the imaging, a dilute solution of the dot sample was deposited onto a silicon oxide-coated copper grid, followed by evaporation to remove the solvent.

Atomic force microscopy (AFM) analysis for the results shown in Figure SM1 was carried out in the acoustic AC mode on a Molecular Imaging PicoPlus AFM system equipped with a multipurpose scanner and a NanoWorld Pointprobe NCH sensor. The height profile analysis was assisted by using the SPIP software distributed by Image Metrology.

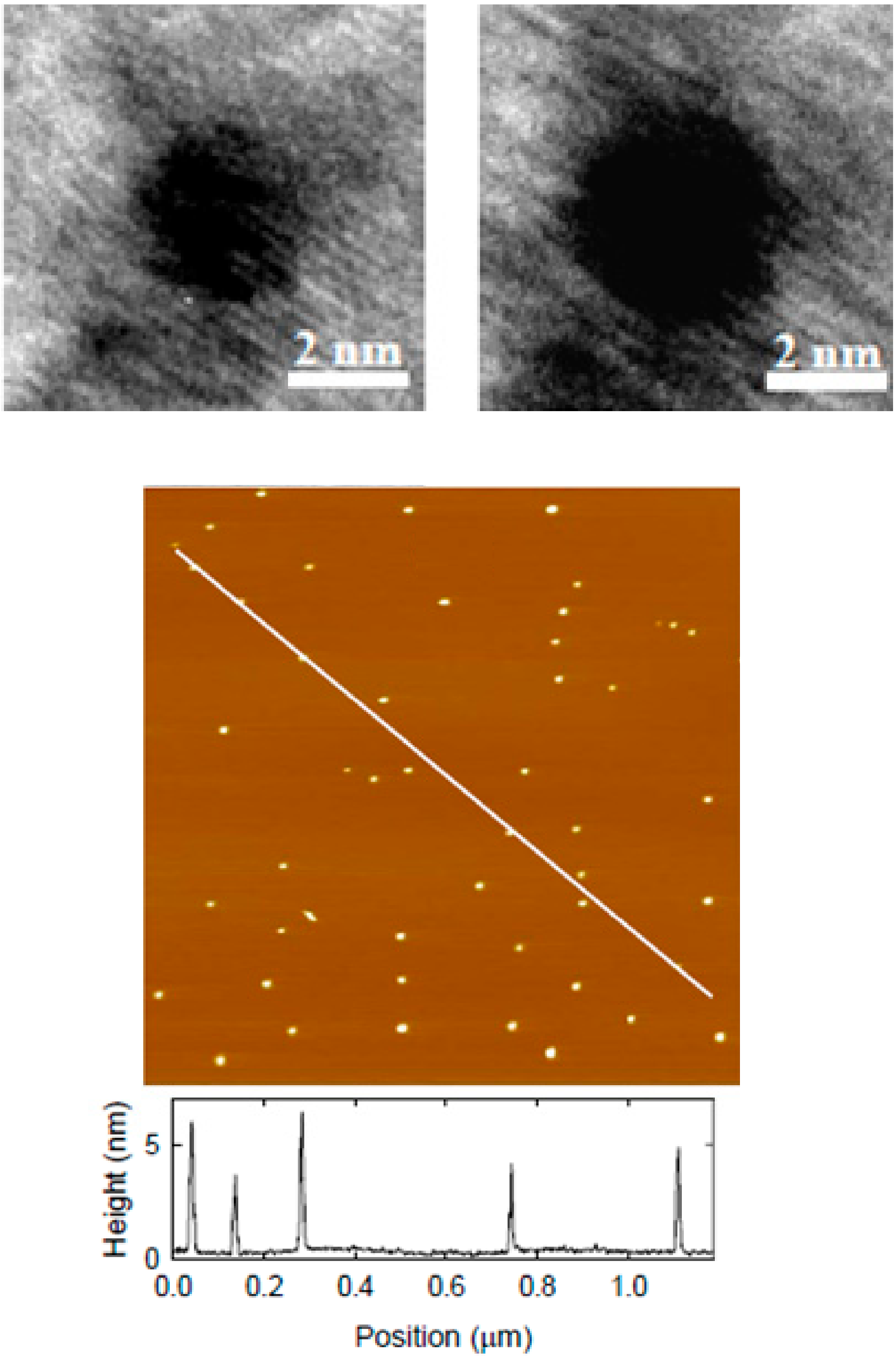

Figure S1. (LOWER) Atomic force microscopy (AFM) topography images of dispersed C_{TiO2}-Dots on mica surface, and (UPPER) corresponding high-resolution transmission electron microscopy (HR-TEM) images of selected individual dots of C_{TiO2}-Dots.
